# Association between comorbid COPD or chronic bronchitis and the prognosis of patients with Dilated cardiomyopathy

**DOI:** 10.1080/07853890.2024.2428857

**Published:** 2024-11-16

**Authors:** Guiting Zhou, Runjia Yu, Chuanjin Luo, Ping Li, Zhihua Huang, Bo Zhang, Guangjiao Liu, Yueqiao Zhong, Jiahua Liang

**Affiliations:** a Cardiovascular Disease, Guangzhou University of Chinese Medicine, Guangzhou, China; b Cardiovascular Disease, Guangzhou University of Traditional Chinese Medicine First Affiliated Hospital, Guangzhou, China; c The Department of Cardiovascular Disease, Meizhou Hospital of Traditional Chinese Medicine, Meizhou, China

**Keywords:** Dilated cardiomyopathy, pulmonary hypertension, chronic obstructive pulmonary disease, chronic bronchitis, prognosis

## Abstract

**Aims:**

Dilated cardiomyopathy (DCM) is characterized by unilateral or bilateral ventricular enlargement and reduced ventricular systolic function, with or without heart failure. In previous studies, we found that a history of chronic obstructive pulmonary disease (COPD) or chronic bronchitis is a high risk factor for DCM combined with pulmonary hypertension (PH). Therefore, we propose that the comorbidity of COPD or chronic bronchitis will increase the cardiogenic mortality of patients with DCM.

**Methods:**

Data were collected from patients with DCM who were admitted to The First Affiliated Hospital of Guangzhou University of Chinese Medicine from October 2008 to April 2020. The primary endpoint was cardiac death. Multivariable Cox regression analyses were employed to assess the associations between the comorbidities COPD or chronic bronchitis with the study endpoints. Different adjusting models were used to adjust for potential confounders.

**Results:**

A total of 305 DCM patients were ultimately enrolled, among whom 46 patients had COPD or chronic bronchitis. The median follow-up was 50 months. The rate of cardiac death in the COPD or chronic bronchitis group was significantly greater than that in the non-COPD or nonchronic bronchitis group (*p* < 0.001). The associations between comorbid COPD or chronic bronchitis and cardiac death remained robust after eliminating the possible effects of confounders. After grouping by PH, the risk difference was mainly derived from the intermediate- or high-probability PH group.

**Conclusions:**

Comorbid COPD or chronic bronchitis increased the risk of cardiac death among DCM patients with an intermediate or high PH probability.

## Introduction

Dilated cardiomyopathy (DCM) is a nonischemic myocardial disease characterized by left ventricular or biventricular dilation and systolic dysfunction without pressure or volume overload or coronary artery disease [1,2]. Patients with DCM present with typical symptoms of systolic heart failure, but as the disease progresses, diastolic dysfunction follows [3]. DCM is a common cause of heart failure, arrhythmia, and sudden death and has a high mortality rate. The aetiology of DCM is complex. Studies have shown that the 5-year survival rate of patients with this disease is less than 50% after the initial diagnosis, and patients have a high mortality rate and poor prognosis [4]. The World Health Organization (WHO) estimated that approximately 5% of all deaths worldwide can be attributed to chronic obstructive pulmonary disease (COPD), which is likely to become the third major cause of death by 2030 [5]. Chronic bronchitis is considered a major component of COPD [6]. However, a study by Franke J et al. revealed that the prevalence of COPD in DCM patients was 36.4% [7].

COPD is a common comorbidity in heart failure patients and can exacerbate the clinical course of the disease, impacting treatment management. A concomitant diagnosis of COPD at the time of heart failure hospitalization increases the risk of subsequent hospitalization by almost 30% [8]. More importantly, related studies have shown that the longer the duration of DCM, the greater the possibility of pulmonary hypertension (PH) caused by a chronic increase in left atrial pressure. More than one-fifth of DCM patients have a high risk of PH [9]. According to previous studies, the incidence of cardiac death is markedly greater in patients with DCM and PH than in DCM patients without PH, with a hazard ratio of 11.79 [10,11]. In previous studies [12], we found that a history of COPD or chronic bronchitis is a high risk factor for DCM combined with PH. Therefore, we propose that the comorbidity of COPD or chronic bronchitis will increase the cardiogenic mortality of patients with DCM.

## Methods

### Patients

Data were collected from patients with DCM who were admitted to The First Affiliated Hospital of Guangzhou University of Chinese Medicine (Guangzhou, Guangdong Province of China) from October 2008 to April 2020. The inclusion criteria were as follows: (1) age > 18 years; (2) a clinical diagnosis of DCM; and (3) a detailed diagnostic work-up, including clinical evaluations, laboratory tests, electrocardiography, and echocardiography. Patients with significant coronary artery disease, primary heart valve disease, restrictive or obstructive cardiomyopathy, congenital heart disease, or severe arterial hypertension were excluded from the study. Echocardiographic assessments were performed on stable patients during outpatient visits or after stabilization in the case of urgent admission. All patients were in stable condition at the time of testing.

Basic information and vital signs for each patient, such as sex, age, systolic blood pressure (BP), diastolic BP, and heart rate, were recorded. The presence of comorbidities, such as hypertension, diabetes mellitus, prior stroke, pulmonary infection, atrial fibrillation, and left bundle branch block (LBBB), was established by medical documentation or in-hospital diagnosis. DCM was defined by the presence of both an LVEF <50% and a dilated LV cavity in the absence of coronary artery stenosis >50% (as determined by coronary angiography), valvular heart disease, arterial hypertension, and secondary cardiac muscle disease attributable to any known systemic condition. The diagnosis of COPD was based on the clinical judgement of the investigator, taking into account the patient’s medical history, treatment and/or spirometric data [8]. The LVEF is an index of left ventricle contractility that indicates the degree of change in the left ventricle volume from diastole to systole. It is calculated by subtracting the end-systolic left ventricle volume from the end-diastolic left ventricle volume and dividing it by the end-diastolic left ventricle volume [13]. We defined chronic bronchitis as the presence of chronic cough and phlegm production that continued for 3 months for more than 2 consecutive years. All patients were followed from the date of their first assessment to the date of cardiac death or death from another cause. The death identification ICD-10 diagnosis codes I00–I09, I11, I13, or I20–I51 were assigned to cardiac death. Patients who died of unknown or other causes, as well as those who had received implanted cardiac resynchronization therapy (CRT) or an implantable cardioverter defibrillator (ICD), were excluded from the analysis. The study protocol complied with the Declaration of Helsinki. Prior to the study, the relevant institutional committees and the First Affiliated Hospital of Guangzhou University of Chinese Medicine Ethical Committee approved the study (protocol number: K[2020]135). Due to the retrospective and anonymous nature of the study, the First Affiliated Hospital of Guangzhou University of Chinese Medicine Ethical Committee waived the requirement for patient informed consent.

### PH probability

The probability of PH was diagnosed in accordance with the 2015 European Society of Cardiology and the European Respiratory Society (ESC/ERS) Guidelines [14]. Briefly, patients were divided into low, intermediate, and high PH probability groups based on echocardiographic assessments at the tricuspid regurgitation peak velocity (TRV) in conjunction with the presence of echocardiographic signs from at least two different categories: (1) pulmonary artery (PA) signs, such as the PA diameter or acceleration time; (2) inferior vena cava (IVC) and right atrium (RA) signs, such as the diameter and inspiratory collapse of the IVC and the RA end-systolic area; and (3) ventricular signs.

### Statistical analysis

Statistical analysis was performed using Stata 15.0 software. (We obtained a copyright licence from Stata 15.0 software.) The data are presented as numbers (percentages). Mann–Whitney U and chi-square (or Fisher’s exact) tests were used for comparisons between groups. Survival curves were constructed using the Kaplan–Meier method, and log-rank tests were used to compare differences between groups. Associations of comorbid COPD or chronic bronchitis with cardiac death were assessed using Cox regression models. We used five different models to adjust for potential confounders. *p* < 0.05 was considered to indicate statistical significance.

## Results

### Baseline characteristics

A total of 305 DCM patients with 71 (23.28%) deaths were ultimately enrolled, among whom 46 patients (15.08%) had COPD or chronic bronchitis as a comorbidity. The characteristics and clinical management during hospitalization of the groups without COPD or chronic bronchitis and with COPD or chronic bronchitis are outlined in Tables 1 and 2. In general, the group of patients with COPD or chronic bronchitis as a comorbidity included more drinkers; those with lower levels of BNP, LVEDd and heart rate; and patients with a greater LVEF.

**Table 1.** Comparison of baseline characteristics between patients with and without COPD or chronic bronchitis.

Characteristics	Without COPD or chronic bronchitis (*n* = 259)	With COPD or chronic bronchitis (*n* = 46)	*p*
Male	182 (70.3)	33 (71.7)	0.840
Age (years)			
≤65	192 (74.1)	29 (63.0)	0.121
>65	67 (25.9)	17 (37.0)	
Duration of DCM (years)			
<1	161 (62.2)	27 (58.7)	
1–5	72 (27.8)	10 (21.7)	0.400
>5	26 (10.0)	9 (19.6)	
Implemented heart failure therapy before hospitalization	69 (26.6)	14 (30.4)	0.594
Systolic BP (mmHg)			
<90	2 (0.8)	1 (2.2)	
90–129	136 (52.5)	21 (45.7)	0.320
≥130	121 (46.7)	24 (52.2)	
Diastolic BP (mmHg)			
<60	8 (3.1)	2 (4.3)	
60–79	72 (27.8)	19 (41.3)	0.146
≥80	179 (69.1)	25 (54.3)	
Heart rate (beats / min)			
<60	6 (2.3)	3 (6.5)	
60–99	140 (54.1)	31 (67.4)	0.039
≥100	113 (43.6)	12 (26.1)	
Ankle edema	135 (52.1)	18 (39.1)	0.104
Smoker	90 (34.7)	22 (47.8)	0.090
Drinker	60 (23.2)	18 (39.1)	0.022
Hypertension classification 1/2/3 (n)	14/43/73	6/7/10	0.581
Diabetes mellitus	66 (25.5)	13 (28.3)	0.692
Prior stroke	16 (6.2)	5 (10.9)	0.400
NYHA class 1/2/3/4 (n)	8/79/103/69	1/15/16/14	0.820
Pulmonary infection	102 (39.4)	14 (30.4)	0.249
Atrial fibrillation/Atrial flutter	64 (24.7)	14 (30.4)	0.412
LBBB	31 (12.0)	6 (13.0)	0.837

*Notes*: Data are presented as *n* (%). *p* < 0.05 is considered statistically significant. *Abbreviations*: COPD, Chronic obstructive pulmonary disease; BP, blood pressure; NYHA, New York Heart Association; LBBB, Left bundle branch block.

**Table 2.** Clinical management during hospitalization.

Characteristics	Without COPD or chronic bronchitis (*n* = 259)	With COPD or chronic bronchitis (*n* = 46)	*p*
Hb (g/L)	139 (125, 150)	132 (114, 151)	0.147
Normal	224 (86.5)	36 (78.3)	
Low	35 (13.5)	10 (21.7)	
PLT (10^9^/L)	201 (160, 249)	196 (160, 254)	0.848
<160	64 (24.7)	11 (23.9)	
160–249	132 (51.0)	22 (47.8)	
>249	63 (24.3)	13 (28.3)	
Leukocyte (10^9^/L)	7.43 (6.04, 9.09)	6.46 (5.73, 8.22)	0.073
<5.91	60 (23.2)	16 (34.8)	
5.91–8.96	129 (49.8)	24 (52.2)	
>8.96	70 (27.0)	6 (13.0)	
NEU (%)	66.3 (58.5, 73.6)	65.4 (58.4, 72.3)	0.667
<58.5	64 (24.7)	11 (23.9)	
58.5–73.5	130 (50.2)	26 (56.5)	
>73.5	65 (25.1)	9 (19.6)	
TC (mmol/L)	4.07 (3.46, 4.74)	4.12 (3.27, 4.74)	0.612
<5.18	217 (83.8)	40 (87.0)	
5.18–6.21	32 (12.4)	4 (8.7)	
>6.21	10 (3.9)	2 (4.3)	
TG (mmol/L)	1.07 (0.82, 1.44)	0.99 (0.79, 1.36)	0.385
<1.70	224 (86.5)	38 (82.6)	
1.70–2.25	19 (7.3)	1 (2.2)	
>2.25	16 (6.2)	7 (15.2)	
LDL-C (mmol/L)	2.71 (2.10, 3.30)	2.68 (2.11, 3.27)	0.946
<3.37	203 (78.4)	36 (78.3)	
3.37–4.13	38 (14.7)	8 (17.4)	
>4.13	18 (6.9)	2 (4.3)	
HDL-C (mmol/L)	0.91 (0.74, 1.15)	0.95 (0.75, 1.17)	0.805
<1.04	164 (63.3)	30 (65.2)	
≥1.04	95 (36.7)	16 (34.8)	
Fasting glucose (mg/dL)	5.1 (4.5, 5.8)	5.1 (4.7, 6.0)	0.418
<3.9	17 (6.6)	1 (2.2)	
3.9–6.1	197 (76.1)	36 (78.3)	
>6.1	45 (17.4)	9 (19.6)	
BNP (pg/mL)	1623 (848, 2570)	653 (346, 1831)	<0.001
<664	53 (20.5)	23 (50.0)	
664–2483	136 (52.5)	17 (37.0)	
>2483	70 (27.0)	6 (13.0)	
Moderate/Severe TR	129 (49.8)	26 (56.5)	0.401
Moderate/Severe MR	168 (64.9)	31 (67.4)	0.740
LVEF (%)	29 (22, 35)	31 (25, 44)	0.020
<22	60 (23.2)	5 (10.9)	
22–36	142 (54.8)	25 (54.3)	
>36	57 (22.0)	16 (34.8)	
LVEDd (mm)	65 (60, 72)	65 (56, 69)	0.013
<60	58 (22.4)	17 (37.0)	
60–71	133 (51.4)	23 (50.0)	
>71	68 (26.3)	6 (13.0)	
TRV (m/s)			0.331
<2.9	133 (51.4)	20 (43.5)	
2.9–3.4	55 (21.2)	11 (23.9)	
>3.4	71 (27.4)	15 (32.6)	
PH probability			0.261
Low	97 (37.5)	14 (30.4)	
Intermediate	71 (27.4)	12 (26.1)	
High	91 (35.1)	20 (43.5)	
Right ventricle diameter (mm)			0.730
<19	59 (22.8)	12 (26.1)	
19–28	151 (58.3)	22 (47.8)	
>28	49 (18.9)	12 (26.1)	
Medication during hospitalization			
Beta-blocker	209 (80.7)	28 (60.9)	0.003
Aldosterone receptor antagonist	224 (86.5)	39 (84.8)	0.757
ACEI or ARB or ARNI	234 (90.3)	39 (84.8)	0.382

*Notes*: Data are presented as medians (interquartile range) and *n* (%). *p* < 0.05 is considered statistically significant. PLT, leukocyte, NEU, BNP, LVEF, LVEDd, and right ventricle diameter were separated into 3 groups according to Quartile. *Abbreviations*: COPD, Chronic obstructive pulmonary disease; Hb, Hemoglobin; PLT, Platelets; NEU, Neutrophil; TC, Total cholesterol; TG, Triglyceride; LDL-C, Low density lipoprotein cholesterol; HDL-C, High density lipoprotein cholesterol; BNP, Brain natriuretic peptide; TR, Tricuspid regurgitation; MR, Mitral regurgitation; LVEF, Left ventricle ejection fraction; LVEDd, Left ventricle end-diastolic diameter; TRV, tricuspid regurgitation peak velocity; PH, pulmonary hypertension; ACEI indicates angiotensin-converting enzyme inhibitor; ARB, angiotensin receptor blocker; ARNI, Angiotensin receptor neprilysin inhibitor.

### Associations between comorbid COPD or chronic bronchitis and clinical outcomes

**Figure 1.**
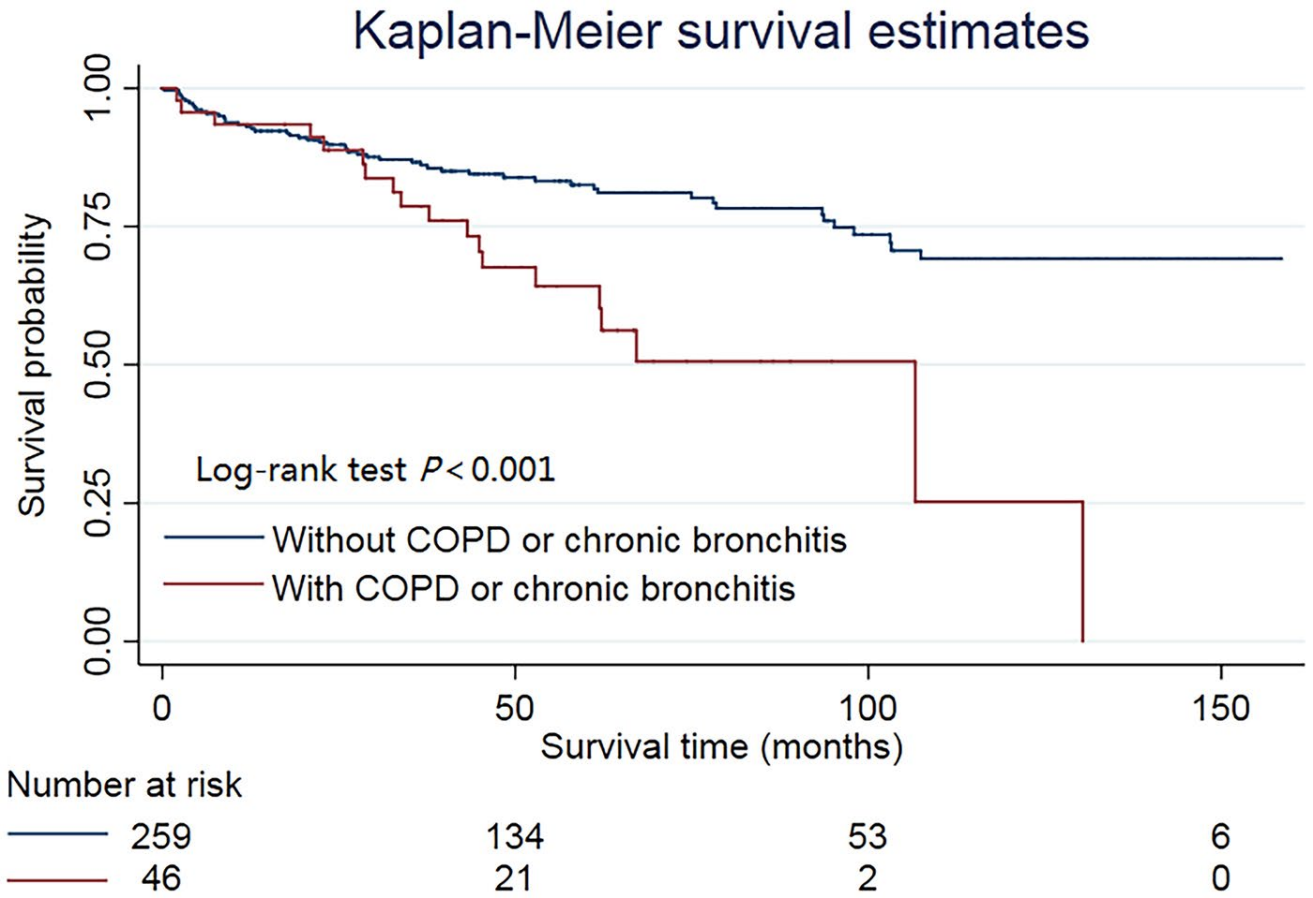
Kaplan-Meier survival curve of the study population. ‘Number at risk’ represents the number of survivors.

The median follow-up was 50 months (interquartile range 28–90 months). The rate of cardiac death in the COPD or chronic bronchitis group was significantly greater than that in the group without COPD or chronic bronchitis (41.3% vs. 20.1%, Table 3). The same result was found in the Kaplan–Meier analysis (Figure 1). We used multivariable Cox regression analysis to eliminate the possible effect of confounders. The results showed that the association remained significant after adjustment for the four different models (HR = 2.998, 95% CI = 1.505–5.974, adjusted by Model 4; the results of the other three models are presented in Table 4).

**Table 3.** Comparison of clinical outcomes.

Characteristics	Without COPD or chronic bronchitis (*n* = 259)	With COPD or chronic bronchitis (*n* = 46)	*p*
Clinical outcomes			
Patients with cardiac death end points	52 (20.1)	19 (41.3)	<0.001*
Patients without cardiac death end points	207 (79.9)	27 (58.7)	

*Notes*: Data are presented as n (%). *Log-rank test. *p* < 0.05 is considered statistically significant. *Abbreviation*: COPD, Chronic obstructive pulmonary disease.

**Table 4.** Association between comorbid COPD or chronic bronchitis and prognosis.

Cardiac death	Group	Hazard ratio	95% CI	*p*
Unadjusted	Without COPD or chronic bronchitis	Reference		0.001
	With COPD or chronic bronchitis	2.514	1.474–4.287	
Adjusted; #1	Without COPD or chronic bronchitis	Reference		0.001
	With COPD or chronic bronchitis	2.455	1.421–4.241	
Adjusted; #2	Without COPD or chronic bronchitis	Reference		0.006
	With COPD or chronic bronchitis	2.201	1.252–3.869	
Adjusted; #3	Without COPD or chronic bronchitis	Reference		<0.001
	With COPD or chronic bronchitis	2.991	1.614–5.544	
Adjusted; #4	Without COPD or chronic bronchitis	Reference		0.002
	With COPD or chronic bronchitis	2.998	1.505–5.974	

*Notes*: Associations of comorbid COPD or chronic bronchitis with cardiac death were analyzed using Cox regression models. The parameters shown in Table 1 and 2 for which *p* < 0.200 and the data of echocardiographic were subjected to multivariable cox regression. *p* < 0.05 is considered statistically significant. #1 -- Demographics (age, smoker, drinker). #2 -- #1 and admission presentation (Diastolic BP, heart rate, ankle edema). #3 -- #2 and serological indicators (Hb, leukocyte, BNP). #4 -- #3 and echocardiographic and medication (Moderate/Severe TR, Moderate/Severe MR, LVEF, LVEDd, Beta-blocker). *Abbreviations*: COPD, Chronic obstructive pulmonary disease; CI, confidence interval; BP, blood pressure; Hb, Hemoglobin; BNP, Brain natriuretic peptide; TR, Tricuspid regurgitation; MR, Mitral regurgitation; LVEF, Left ventricle ejection fraction; LVEDd, Left ventricle end-diastolic diameter.

**Figure 2.**
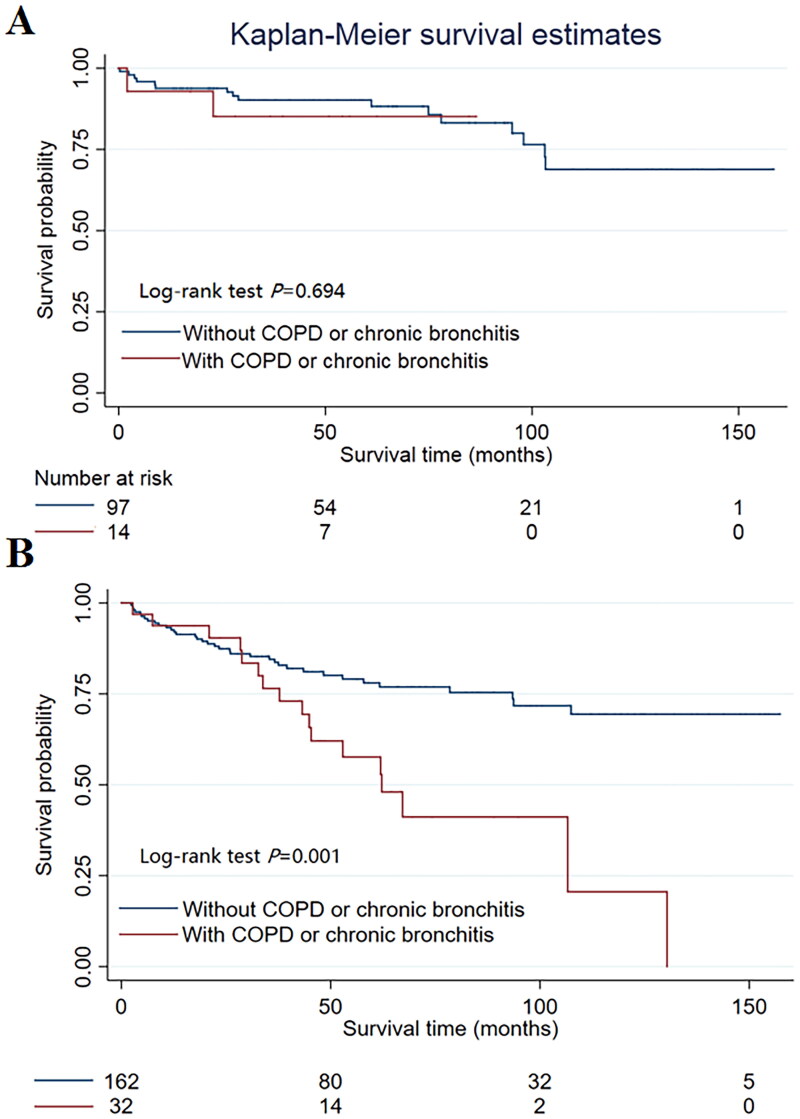
The Kaplan-Meier analysis in the subgroup of PH probability. (A) In the low PH probability subgroup, COPD or chronic bronchitis did not affect the survival rate (*p* = 0.694). (B) In the subgroup of intermediate or high PH probability, patients with COPD or chronic bronchitis had a lower survival rate than patients without COPD or chronic bronchitis (*p* = 0.001).

After a subgroup analysis stratified according to PH probability, in the low PH probability subgroup, COPD or chronic bronchitis did not affect the survival rate (HR = 1.350, 95% CI = 0.301–6.061; *p* = 0.695). In the subgroup with an intermediate or high PH probability (HR = 2.645, 95% CI = 1.477–4.737, *p* = 0.001), patients with COPD or chronic bronchitis had a lower survival rate than patients without COPD or chronic bronchitis (as presented in Table 5 and Figure 2).

**Table 5.** Association of comorbid COPD or chronic bronchitis with the prognosis subgroup analysis by PH probability.

Cardiac death	Group	Hazard ratio	95% CI	*p*
PH probability				
Low	Without COPD or chronic bronchitis	Reference		0.695
	With COPD or chronic bronchitis	1.350	0.301–6.061	
Intermediate or high	Without COPD or chronic bronchitis	Reference		0.001
	With COPD or chronic bronchitis	2.645	1.477–4.737	

*Notes*: Association of Comorbid COPD or chronic bronchitis with prognosis subgroup analysis by PH probability were analyzed using Cox regression models. *p* < 0.05 is considered statistically significant. *Abbreviations*: COPD, Chronic obstructive pulmonary disease; CI, confidence interval; PH, pulmonary hypertension.

## Discussion

To the best of our knowledge, this study is the first to explore the effect of COPD or chronic bronchitis on cardiac mortality in patients with DCM. In this study, 305 DCM patients were selected for a retrospective analysis and divided into low-, intermediate- and high-probability PH groups. The results showed that the risk of cardiac death in patients with DCM was greater when DCM was combined with COPD or chronic bronchitis. After grouping according to PH, the aforementioned risk difference was mainly reflected in the intermediate- or high-probability PH group.

The Kaplan–Meier survival curve revealed that COPD or chronic bronchitis significantly reduced the survival rate of DCM patients, and the difference was more obvious at longer survival times. We used Cox regression models to minimize the deviation caused by confounding factors, and the results were the same. We propose two hypotheses to explain the results. First, patients with COPD or chronic bronchitis had a greater risk of PH. Second, patients with COPD have lower utilization of beta-blockers.

First, the occurrence of COPD is irreversible, characterized by airflow limitation and caused by harmful gases or particles entering the body [15]. Chronic bronchitis is a common chronic respiratory disease that is characterized by recurrent cough, expectoration or a chronic course with wheezing [16]. COPD generally develops from chronic bronchitis and emphysema with a progressive, incompletely reversible limitation of airflow caused by a mixture of small airway disease and gas exchange impairment through parenchymal destruction [5,17,18]. COPD and chronic bronchitis have long disease courses and easily relapse, making them difficult to cure; they are also associated with the long-term use of antibiotics, bronchodilators and other drugs, which can have major side effects. Most of the patients are elderly individuals whose physical function is reduced and whose body resistance is low. PH can be triggered by hyperinflation, airway obstruction and airway collapse in patients with advanced COPD [19]. However, PH is directly related to the prognosis of DCM patients, and the incidence of cardiac death is markedly greater in patients with DCM and PH than in DCM patients without PH [11]. The development of PH in DCM is a multistage process. Left ventricular dilatation in patients with DCM leads to left ventricular systolic or diastolic dysfunction, resulting in MR. Subsequently, due to the increase in left ventricle filling pressure and passive backwards transmission, the left atrial pressure shows a chronic increasing trend, thereby increasing the pressure of the pulmonary veins and pulmonary arteries. As PH progresses, pulmonary endothelial dysfunction leads to obvious vasoconstriction and irreversible remodelling of pulmonary vessels, resulting in right ventricular deposition and hypertrophy. Because the right ventricle is more sensitive to long-term pressure overload, over time, the right side of the heart may be irreversibly damaged, eventually leading to heart failure. Due to the gradual expansion of the heart, reduced ventricular systolic function, and arrhythmia, DCM patients are often hospitalized repeatedly for heart failure. Some patients also have PH and haemodynamic changes as the disease progresses, resulting in right ventricular remodelling, causing heart failure and evolving to the final stage.

Regarding the second explanation, with the continuous advancement of medical science, the benefits of pharmacological and nonpharmacological treatments for patients with DCM combined with COPD have become more obvious [20]. Beta-blockers have efficacy against some cardiovascular diseases. They are considered conventional drugs for the treatment of heart failure and can reduce pulmonary artery pressure. CVDs often coexist with pulmonary diseases [20], especially COPD. Due to concerns about adverse reactions such as the contraction of bronchial smooth muscle, especially in patients with advanced chronic COPD, beta-blockers have not been widely used in COPD patients [21,22]. Our study revealed a significant disparity in beta-blocker use, with lower rates in the COPD and chronic bronchitis groups, as shown in Table 2. This result is concerning because a higher heart rate is independently associated with greater mortality in both COPD and heart failure patients [23,24]. Notably, most of the data were obtained from populations enrolled more than a decade ago, when the importance of neurohormonal inhibition of the sympathetic nervous system in heart failure was less recognized. Therefore, our findings underscore the ongoing challenge of appropriately treating patients with DCM with COPD, which warrants greater attention from the cardiology community.

Our study showed that patients with DCM combined with COPD or chronic bronchitis had a greater risk of cardiac death. After stratification by PH, a greater risk of cardiac death was observed mainly in the intermediate- or high-probability PH group, while no significant difference was noted in the low-PH probability group. According to the 2022 European Guidelines for Pulmonary Hypertension [25], in symptomatic patients with an intermediate or high echocardiographic probability of PH, further testing may be necessary to further determine the likelihood of PH and develop management strategies. However, although right heart catheterization (RHC) is the gold standard for PH diagnosis, it is relatively complicated, expensive, and invasive and is associated with a number of complications [26]. Therefore, most patients do not choose further diagnosis, resulting in insufficient management of PH during the follow-up period. As we mentioned earlier, the development of PH is a multistage process in the course of DCM. COPD or chronic bronchitis can affect the occurrence and progression of PH. Therefore, in the intermediate- or high-probability PH group, COPD or chronic bronchitis can accelerate the progression of PH, which is significantly related to the survival rate of patients with PH. Similarly, since PH is directly related to the prognosis of DCM patients, the prognosis is likely affected by COPD or chronic bronchitis, causing this difference in risk.

This study has several limitations. First, many changes may have been made in heart failure and COPD treatment after discharge. We do not have specific data on the treatment that the patients received or on changes in pulmonary artery pressure during this time period. Second, as this study was retrospective and not all patients underwent pulmonary function tests during hospitalization, we were unable to assess the severity of COPD. Third, we did not perform RHC for PH diagnosis. Although RHC is an invasive and expensive examination, as the gold standard for the diagnosis of PH, it is undoubtedly more accurate for the determination of parameters related to PH. Fourth, the sample size was small, and bias may exist in the results of the subgroup analysis. Moreover, we included only patients with relatively complete baseline information and available follow-up data, indicating selection bias in the study population, which somewhat affected the validity of the statistical analysis. Multicentre, large-sample studies are needed to confirm our results.

## Conclusions

Comorbid COPD or chronic bronchitis was associated with an increased incidence of cardiac death in DCM patients. After grouping by PH, the risk difference was mainly derived from the intermediate- or high-probability PH group. Our findings underscore the ongoing challenge of appropriately treating patients with DCM along with COPD, and in patients with an intermediate or high echocardiographic probability of PH, RHC is necessary for the further diagnosis of PH and development of management strategies.

## Data Availability

The data that support the findings of this study are available from the corresponding author upon reasonable request.

## References

[1] Schultheiss H, Fairweather D, Caforio ALP, et al. Dilated cardiomyopathy. Nat Rev Dis Primers. 2019;5(1):32. doi: 10.1038/s41572-019-0088-x. 31073128 PMC7096917

[2] Sinagra G, Elliott PM, Merlo M. Dilated cardiomyopathy: so many cardiomyopathies! Eur Heart J. 2020;41(39):3784–3786. doi: 10.1093/eurheartj/ehz908. 31872205

[3] Reichart D, Magnussen C, Zeller T, et al. Dilated cardiomyopathy: from epidemiologic to genetic phenotypes: a translational review of current literature. J Intern Med. 2019;286(4):362–372. doi: 10.1111/joim.12944. 31132311

[4] Japp AG, Gulati A, Cook SA, et al. The diagnosis and evaluation of dilated cardiomyopathy. J Am Coll Cardiol. 2016;67(25):2996–3010. doi: 10.1016/j.jacc.2016.03.590. 27339497

[5] Teo E, Lockhart K, Purchuri SN, et al. Haemophilus influenzae oral vaccination for preventing acute exacerbations of chronic bronchitis and chronic obstructive pulmonary disease. Cochrane Database Syst Rev. 2017;6(6):CD010010. doi: 10.1002/14651858.CD010010.pub3. 28626902 PMC6481520

[6] Bhatt SP, Bodduluri S, Kizhakke Puliyakote AS, et al. Structural airway imaging metrics are differentially associated with persistent chronic bronchitis. Thorax. 2021;76(4):343–349. doi: 10.1136/thoraxjnl-2020-215853. 33408194 PMC8225550

[7] Franke J, Zugck C, Hochadel M, et al. Etiology-specific assessment of predictors of long-term survival in chronic systolic heart failure. Int J Cardiol Heart Vasc. 2015;7:61–68. doi: 10.1016/j.ijcha.2015.01.015. 28785647 PMC5497234

[8] Canepa M, Straburzynska-Migaj E, Drozdz J, et al. Characteristics, treatments and 1-year prognosis of hospitalized and ambulatory heart failure patients with chronic obstructive pulmonary disease in the European Society of Cardiology Heart Failure Long-Term Registry. Eur J Heart Fail. 2018;20(1):100–110. doi: 10.1002/ejhf.964. 28949063

[9] Dziewięcka E, Wiśniowska-Śmiałek S, Karabinowska A, et al. Relationships between pulmonary hypertension risk, clinical profiles, and outcomes in dilated cardiomyopathy. J Clin Med. 2020;9(6):1660. doi: 10.3390/jcm9061660. 32492830 PMC7355437

[10] Hirashiki A, Kondo T, Adachi S, et al. Prognostic value of pulmonary hypertension in ambulatory patients with non-ischemic dilated cardiomyopathy. Circ J. 2014;78(5):1245–1253. doi: 10.1253/circj.cj-13-1120. 24621657

[11] Vachiéry JL, Tedford RJ, Rosenkranz S, et al. Pulmonary hypertension due to left heart disease. Eur Respir J. 2019;53(1):1801897. doi: 10.1183/13993003.01897-2018. 30545974 PMC6351334

[12] Liang J, Zhu R, Yang Y, et al. A predictive model for dilated cardiomyopathy with pulmonary hypertension. ESC Heart Fail. 2021;8(5):4255–4264. doi: 10.1002/ehf2.13535. 34338447 PMC8497218

[13] Fukuda S, Watanabe H, Daimon M, et al. Normal values of real-time 3-dimensional echocardiographic parameters in a healthy Japanese population: the JAMP-3D Study. Circ J. 2012;76(5):1177–1181. doi: 10.1253/circj.cj-11-1256. 22361920

[14] Galiè N, Humbert M, Vachiery JL, et al. 2015 ESC/ERS Guidelines for the Diagnosis and Treatment of Pulmonary Hypertension. Rev Esp Cardiol. 2016;69(2):177. doi: 10.1016/j.rec.2016.01.002. 26837729

[15] Numata T, Nakayama K, Fujii S, et al. Risk factors of postoperative pulmonary complications in patients with asthma and COPD. BMC Pulm Med. 2018;18(1):4. doi: 10.1186/s12890-017-0570-8. 29316890 PMC5761153

[16] Balte PP, Chaves PHM, Couper DJ, et al. Association of nonobstructive chronic bronchitis with respiratory health outcomes in adults. JAMA Intern Med. 2020;180(5):676–686. doi: 10.1001/jamainternmed.2020.0104. 32119036 PMC7052787

[17] Kesimer M, Ford AA, Ceppe A, et al. Airway mucin concentration as a marker of chronic bronchitis. N Engl J Med. 2017;377(10):911–922. doi: 10.1056/NEJMoa1701632. 28877023 PMC5706541

[18] Lahousse L, Seys LJM, Joos GF, et al. Epidemiology and impact of chronic bronchitis in chronic obstructive pulmonary disease. Eur Respir J. 2017;50(2):1602470. doi: 10.1183/13993003.02470-2016. 28798087 PMC5593375

[19] Pichl A, Sommer N, Bednorz M, et al. Riociguat for treatment of pulmonary hypertension in COPD: a translational study. Eur Respir J. 2019;53(6):1802445. doi: 10.1183/13993003.02445-2018. 30956210

[20] Dézsi CA, Szentes V. The real role of β-blockers in daily cardiovascular therapy. Am J Cardiovasc Drugs. 2017;17(5):361–373. doi: 10.1007/s40256-017-0221-8. 28357786 PMC5591796

[21] Salpeter S, Ormiston T, Salpeter E. Cardioselective beta-blockers for chronic obstructive pulmonary disease. Cochrane Database Syst Rev. 2005;2005(4):CD003566. doi: 10.1002/14651858.CD003566.pub2. 16235327 PMC8719355

[22] Lipworth B, Skinner D, Devereux G, et al. Underuse of β-blockers in heart failure and chronic obstructive pulmonary disease. Heart. 2016;102(23):1909–1914. doi: 10.1136/heartjnl-2016-309458. 27380949 PMC5136686

[23] Jensen MT, Marott JL, Lange P, et al. Resting heart rate is a predictor of mortality in COPD. Eur Respir J. 2013;42(2):341–349. doi: 10.1183/09031936.00072212. 23143550

[24] Dobre D, Borer JS, Fox K, et al. Heart rate: a prognostic factor and therapeutic target in chronic heart failure. The distinct roles of drugs with heart rate-lowering properties. Eur J Heart Fail. 2014;16(1):76–85. doi: 10.1093/eurjhf/hft129. 23928650

[25] Humbert M, Kovacs G, Hoeper MM, et al. 2022 ESC/ERS Guidelines for the diagnosis and treatment of pulmonary hypertension. Eur Heart J. 2022;43(38):3618–3731. [published correction appears in Eur Heart J. 2023 Apr 17;44(15):1312]. doi: 10.1093/eurheartj/ehac237. 36017548

[26] Vormbrock J, Liebeton J, Wirdeier S, et al. Determinants of right ventricular muscle mass in idiopathic dilated cardiomyopathy: impact of left ventricular muscle mass and pulmonary hypertension. Int J Med Sci. 2014;11(8):834–840. doi: 10.7150/ijms.6961. 24936147 PMC4057489

